# Surgical Treatment of Vertebral Compression Fracture in a Patient with Idiopathic Normal Pressure Hydrocephalus

**DOI:** 10.31662/jmaj.2023-0005

**Published:** 2023-06-12

**Authors:** Tatsuya Tanaka, Ren Fujiwara, Ryohei Sashida, Yu Hirokawa, Tomihiro Wakamiya, Yuhei Michiwaki, Kazuaki Shimoji, Eiichi Suehiro, Keisuke Onoda, Fumitaka Yamane, Masatou Kawashima, Akira Matsuno

**Affiliations:** 1 Department of Neurosurgery, International University of Health and Welfare, School of Medicine Narita Hospital, Narita, Japan; 2 Department of Neurosurgery, Narita Tomisato Tokushukai Hospital, Tomisato, Japan

**Keywords:** vertebral compression fracture, balloon kyphoplasty, idiopathic normal pressure hydrocephalus, lumboperitoneal shunt, risk factor, quality of life

## Abstract

Idiopathic normal pressure hydrocephalus (iNPH) with gait disturbance can be effectively treated with a cerebrospinal fluid shunt. Furthermore, balloon kyphoplasty (BKP) is a successful minimally invasive treatment for osteoporotic vertebral compression fractures (VCFs). This case report presents the surgical management of an elderly patient with iNPH who presented after a VCF due to a fall. A 77-year-old woman who had been experiencing progressive gait disturbance for five years reported experiencing back pain one month after a fall. Imaging revealed a recent L1 VCF that did not compromise the spinal canal. Furthermore, the Mini-Mental State Examination results and the timed up-and-go test were 20 points and 17.96 seconds, respectively. Magnetic resonance imaging revealed ventriculomegaly with an Evans’ index of 0.35. Her symptoms improved temporarily after a tap test, and she was diagnosed with probable iNPH. BKP was performed for VCFs, followed by the lumboperitoneal (LP) shunt placement for iNPH one month later. Following the operation, her symptoms improved without complications. After one month of performing BKP, an LP shunt would be placed to prevent shunt complications, such as infection and catheter-related neurological symptoms. Screening for iNPH in the elderly who present after VCFs due to a fall may identify iNPH patients who may benefit more from surgical treatments.

## Introduction

Idiopathic normal pressure hydrocephalus (iNPH) is a progressive syndrome characterized by gait disturbance, cognitive impairment, and urinary incontinence, including ventricular enlargement on neurological imaging and normal cerebrospinal fluid (CSF) pressure ^(1)^. Several studies have reported fall rates of 56%-82% before iNPH diagnosis ^(2), (3)^. Further, it has been reported that 7.7% of patients with hip fracture have possible iNPH ^(4)^. Therefore, patients with iNPH may be relatively more prone to fractures due to falls.

The prevalence rates of vertebral compression fractures (VCFs) in participants aged 70 and ≥80 years are reported to be 26.3% and 41.5% in men and 27.1% and 53.0% in women, respectively ^(5)^. VCFs are a global burden that could temporarily or permanently affect millions of elderly people. Balloon kyphoplasty (BKP) is a widely accepted minimally invasive surgical technique for treating osteoporotic VCFs ^(6)^. This case report presents the case of an elderly patient who was diagnosed with iNPH following a VCF caused by a fall. To the best of our knowledge, this report pioneers describing surgical management in a patient with iNPH and VCF.

## Case Report

A 77-year-old woman with progressive gait difficulty, urge incontinence, and cognitive impairment for five years reported experiencing back pain one month after a fall.

**Figure 1.**
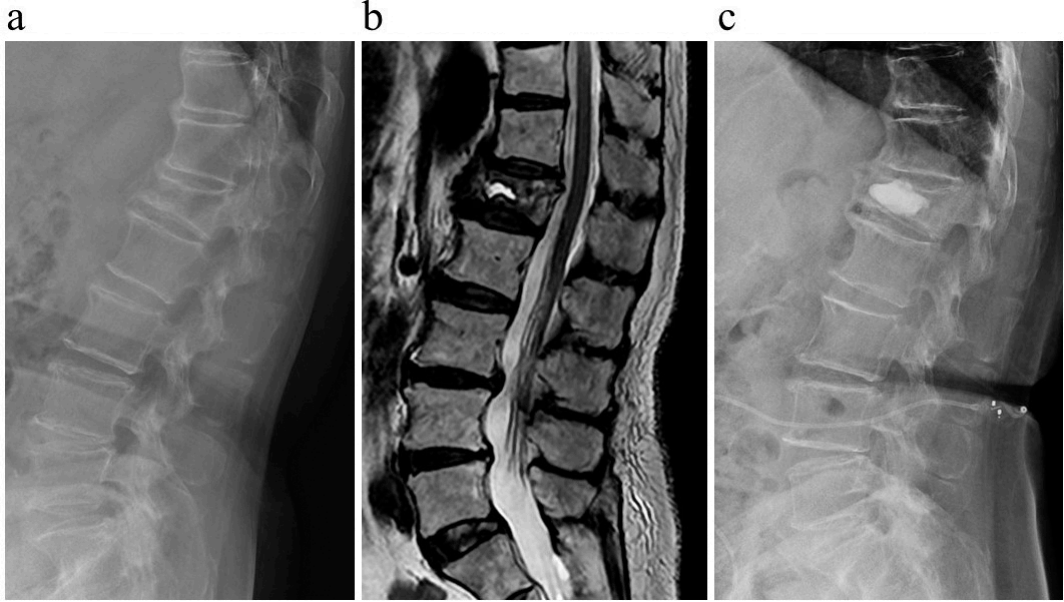
Preoperative plain spinal radiographs demonstrated L1 compression fracture (a). T2-weighted imaging on magnetic resonance imaging revealed a recent L1 vertebral compression fracture without spinal canal compromise (b). Plain spinal radiographs after balloon kyphoplasty reveal effective filling of cystic fracture cavity (c).

Standing and walking aggravated the pain, which was relieved by rest. Physical examination revealed pain when we knocked the patient’s midback. There was no evidence of a neurologic deficit. Initially, she received conservative treatment, but it was inadequate. Plain spinal radiographs demonstrated L1 compression fracture (Figure 1a). Computed tomography showed a collapse fracture of the L1 vertebrae, and T2-weighted imaging on magnetic resonance imaging revealed a recent L1 VCF without spinal canal compromise (Figure 1b).

**Figure 2.**
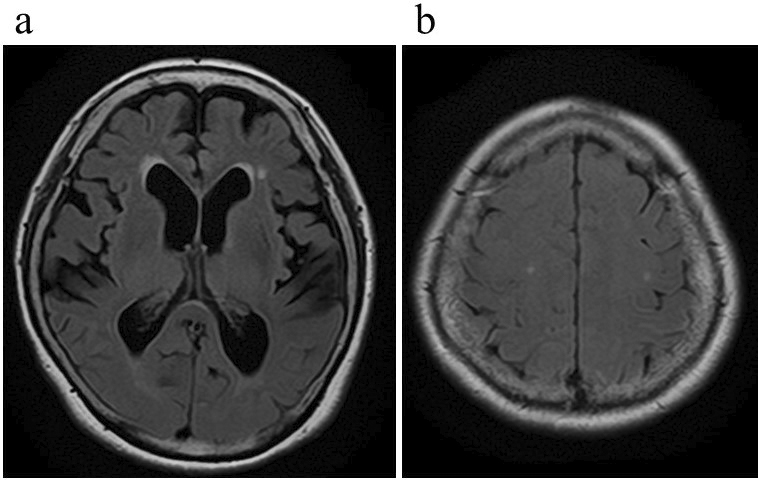
Magnetic resonance imaging showed disproportionately enlarged subarachnoid space hydrocephalus, such as ventriculomegaly with an Evans’ index of 0.35 (a), narrowing of the cerebrospinal fluid spaces near the vertex, and widening of the Sylvian fissure (b).

Furthermore, the Mini-Mental State Examination results and the timed up-and-go test were 20 points and 17.96 seconds, respectively. The total score of the iNPH grading scale (iNPHGS) was 5, with the following domain scores: gait disturbance (g), 2; cognitive impairment (c), 3; and urinary disturbance (u), 0. Magnetic resonance imaging showed disproportionately enlarged subarachnoid space hydrocephalus, such as ventriculomegaly with an Evans’ index of 0.35, narrowing of the CSF spaces near the vertex, and widening of the Sylvian fissure (Figure 2). Her symptoms improved temporarily after a tap test, and she was diagnosed with probable iNPH.

The patient had BKP for an L1 compression fracture. During the surgery, under fluoroscopic guidance, bone access needles consisting of cannula tubes and stylets were introduced into the bilateral pedicles at the accurate spinal level: the L1 level and a working tunnel were created. Then, the balloon was delivered through a bilateral working tunnel to elevate and restore the vertebral body height. Consequently, cement was injected. BKP was performed (Figure 1c), resulting in pain relief, allowing her to resume her daily activities.

One month after BKP, the patient underwent lumboperitoneal (LP) shunt placement for iNPH.

**Figure 3.**
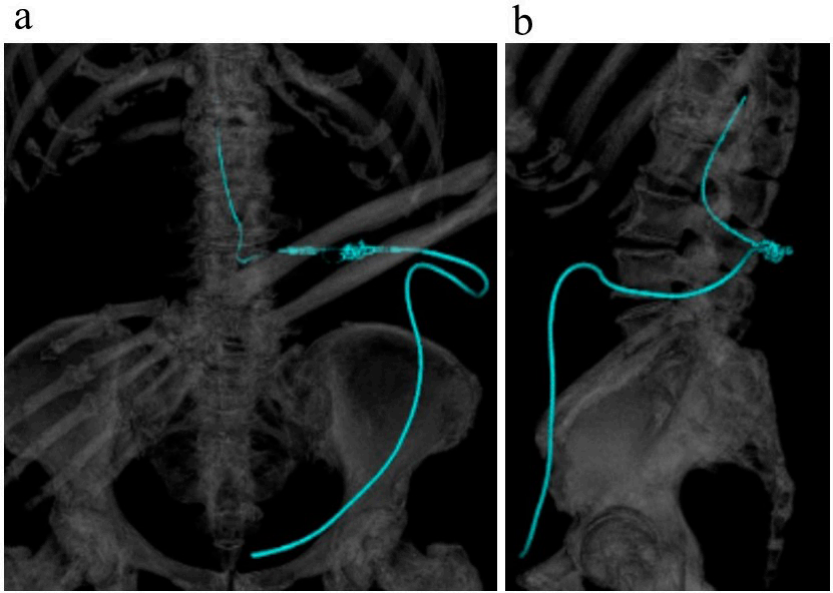
Postoperative three-dimensional computed tomography showed the programmable valve and catheter’s correct placement.

LP shunt placement using the Certas™ Plus Programmable Valve was performed. The programmable valve and catheter were correctly placed according to postoperative three-dimensional computed tomography (Figure 3). After the operation, her symptoms improved and iNPHGS was 3 (g 1; c 2; u 0). After six months of follow-up, there was no recurrence of fractures.

## Discussion

Among the common clinical features of iNPH, gait disturbance is the most common initial symptom. According to Nikaido et al., 82.6% of iNPH patients had a history of falls in the six months preceding shunt surgery ^(2)^, demonstrating that falls are hallmark symptoms of iNPH. The prevalence of possible iNPH has been reported to be 1.4%-3.7% in the general population aged 65 years or older ^(7), (8)^. Moreover, a recent study reported that 18.7% of patients aged 60-89 years who presented after a fall had possible iNPH, 12.3% had probable iNPH, and 10.6% had definite iNPH ^(9)^. Furthermore, it has been reported that 7.7% of patients with hip fracture had possible iNPH, and a relationship between hip fractures and iNPH-related falls has been suggested previously ^(4)^. Previous reports show that the prevalence of iNPH in VCF patients is unknown. Therefore, screening elderly patients with VCFs due to falls for a history of suspected iNPH, such as gait disturbance, dementia, and urinary incontinence, is crucial as early detection and treatment of iNPH in such patients may prevent further incidences of fracture.

LP shunt placement one month after BKP aims to prevent shunt complications like infection and catheter-related neurological symptoms. Concerning the time interval between percutaneous endoscopic gastrostomy tube and ventriculoperitoneal (VP) shunt insertion, Nabika et al. insisted on a 1-month interval ^(10)^. Naylor et al. hypothesized that a 6-week interval between shunt construction and posterior cervical surgery would adequately manage the risk of infection and provide these patients sufficient time to recover physically ^(11)^. Although the risk of infection after BKP is extremely low, LP shunt placement was performed one month after BKP to prevent any chances of shunt infection. Sato et al. reported that kyphosis caused by VCF at L1 contributed to the lumber catheter becoming tangled ^(12)^. To prevent catheter-related neurological symptoms after spinal surgery, LP shunts or VP shunts should be performed.

This case report presents a patient with iNPH, VCF, and surgical management. After one month of performing BKP, an LP shunt could be placed to prevent shunt complications, such as infection and catheter-related neurological symptoms. Screening for iNPH in the elderly patients who present after VCFs due to a fall may identify iNPH patients who may benefit more from surgical treatments.

## Article Information

### Conflicts of Interest

None

### Author Contributions

Tatsuya Tanaka: care of patients and writing, designing, and manuscript editing. Ren Fujiwara, Ryohei Sashida, Yu Hirokawa, Tomihiro Wakamiya, and Yuhei Michiwaki: care of patients and manuscript editing. Kazuaki Shimoji, Eiichi Suehiro, Keisuke Onoda, Fumitaka Yamane, Masatou Kawashima, and Akira Matsuno: manuscript editing.

### Informed Consent

The patient had agreed and signed informed consent regarding publishing the case in an academic journal without exposing his identity.

### Approval by Institutional Review Board (IRB)

Not applicable
